# Global, regional, and national disease burden of tobacco-related Alzheimer’s disease among individuals over the age of 55: a global burden of disease study

**DOI:** 10.3389/fpubh.2025.1581871

**Published:** 2025-05-14

**Authors:** Tianyi Dong, Shipeng Zhang, Hanyu Wang, Yanjie Jiang, Qinxiu Zhang, Xueying Li, Lanfang He

**Affiliations:** ^1^Hospital of Chengdu University of Traditional Chinese Medicine, Chengdu University of Traditional Chinese Medicine, Chengdu, China; ^2^Nanjing Hospital of Chinese Medicine Affiliated to Nanjing University of Chinese Medicine, Nanjing, China; ^3^World Health Organization Collaborating Centre (WHOCC), CHN-56, Chengdu, China; ^4^Department of Ultrasound, Hospital of Chengdu University of Traditional Chinese Medicine, Chengdu, China

**Keywords:** global burden of disease, Alzheimer’s disease, tobacco use, risk factor, ASDR

## Abstract

**Background:**

Alzheimer’s disease is a progressive neurodegenerative disorder characterized by an insidious onset. Numerous studies have identified a significant association between tobacco use and Alzheimer’s disease. This study aims to explore the epidemiological patterns and trends concerning tobacco-related Alzheimer’s disease at global, national and regional levels.

**Methods:**

We analyzed data on mortality, age-standardized DALY rate (ASDR), and estimated annual percentage changes (EAPCs) sourced from the Global Burden of Disease data for 2021. The analysis was further stratified by country and region, socio-demographic index (SDI), gender, and age. A Bayesian Age-Period-Cohort (BAPC) model was employed to project the global burden in the future.

**Results:**

In 2021, the total burden revealed a decline in the number of deaths and ASDR compared to 1990. The highest proportions of mortality and ASDR were observed in the age group over 95 years. The disease burden among men was significantly higher than of among women, approximately three times greater. Conversely, in Australia and North America, the burden of disease among women surpassed that of men. In most of the 21 regions worldwide, both mortality and ASDRs have decreased since 1990, and intra-regional mortality rates have declined as SDI has increased. It is anticipated that the burden will continue to gradually decrease from 2021 to 2040.

**Conclusion:**

Although the global burden of tobacco-related Alzheimer’s disease among the older adults declined from 1990 to 2021, significant disparities existed across regions, age groups, sex, and SDI.

## 1 Introduction

Alzheimer’s disease (AD) is a progressive degenerative disease of nervous system with hidden onset (1). The primary clinical manifestations in the early stages include short-term memory loss, cognitive deficits, and affective impairments. In the later stages, individuals may experience limb muscle paralysis and a loss of self-care abilities. Dementia currently ranks as the seventh leading cause of death globally and is one of the primary contributors to disability and dependence among older adults (2). It is estimated that over 55 million individuals worldwide are affected by dementia, with Alzheimer’s disease representing 60 to 70% of these cases (3). The global economic burden of the disease has reached 13.5 trillion US dollars and is projected to continue increasing. By 2050, the number of affected individuals is expected to rise to 139 million, with the most significant increases occurring in low- and middle-income countries (3).

Cigarettes are tobacco products characterized by nicotine as their primary component, and the smoke produced by burning cigarettes contains numerous carcinogenic substances. The World Health Organization asserts that all forms of tobacco use are detrimental to health, with no safe level of exposure to tobacco (4). Cigarettes have been unequivocally identified as a significant risk factor for various diseases, including oral cancer, esophageal cancer, hypertension, and coronary heart disease (5). Furthermore, smoking is recognized on the official website of the International Alzheimer’s Association as one of the twelve prominent risk factors for Alzheimer’s disease (6), with numerous studies establishing a significant correlation between both past and current smoking behaviors and the risk of developing Alzheimer’s disease (7–9). Numerous studies have demonstrated a positive association between smoking and the risk and incidence of Alzheimer’s disease (10, 11). However, the epidemiological patterns and trends concerning tobacco-related Alzheimer’s disease remain largely unexplored at global, national, and regional levels. Therefore, to warrant the detriment cigarette does to AD, and to optimize regional health policies, this is the first article to investigate various indicators and trends of tobacco-related Alzheimer’s disease from a global perspective. As a neurodegenerative disorder, Alzheimer’s disease predominantly affects middle-aged and older adults individuals. This article aims to explore the global burden of Alzheimer’s disease among individuals over 55 years of age by analyzing data from the 2021 Global Burden of Disease (GBD) database, thereby providing a valuable reference for government decision-making, medical research, and clinical risk assessment.

## 2 Methods

### 2.1 Overview and definition

The GBD 2021 study provides extensive data on the burden of 359 diseases and injuries, as well as 84 risk factors, across 204 countries and territories worldwide from 1990 to 2021. These countries and regions are categorized into 21 regions and 5 groups based on the Socio-Demographic Index (SDI). The GBD study estimated exposure levels and trends, attributable deaths, and attributable disability-adjusted life years (DALY) for 84 risk factors, disaggregated by age group, sex, year, and location (12). The assessed risk factors encompass metabolic, occupational, environmental, and behavioral dimensions. DALY, or Disability-Adjusted Life Years, comprises both years lost due to premature death (YLL) and years lived with disability (YLD). Alzheimer’s disease is a progressive, neurodegenerative disease that seriously affects the quality of life in later life. According to the International Classification of Diseases (ICD), the classification code for Alzheimer’s disease is 8A20.

### 2.2 Estimation of tobacco exposure

The risk factors for tobacco exposure in the Global Burden of Disease (GBD) database are categorized under behavioral factors, which include both current and past histories of smoking tobacco products, the use of chewing tobacco, and passive exposure to secondhand smoke. Active tobacco exposure was quantified by the number of cigarettes smoked per day and the cumulative years of smoking. In the social and human sciences, smoking exposure is assessed using self-reported data from major representative survey series, including household composition modules such as the Demographic and Health Surveys (DHS), Multiple Indicator Cluster Surveys (MICS), and Living Standards Measurement Surveys (LSMS), as well as national and local censuses. Additionally, census data from the IPUMS project were incorporated, with all data being sourced from the Global Health Data Exchange Directory (GHDx). The GBD database employed a Comparative Risk Assessment (CRA) framework to evaluate the burden of Alzheimer’s disease attributable to tobacco exposure (12). We utilized GBD result generation tools at the Institute for Health Metrics and Evaluation to obtain data on the burden of tobacco-related Alzheimer’s disease, expressed in terms of the number of deaths, age-standardized mortality rate (ASMR), disability-adjusted life years (DALY), and age-standardized DALY rate (ASDR) by country, region, sex, age, and Socio-Demographic Index (SDI).^1^ ASMR and ASDR are generally regarded as more accurate and reliable epidemiological indicators for comparing disease burden, as they account for variations in age structure.

### 2.3 Statistical analysis and visualization process

We calculated the estimated annual percentage change (EAPC) to assess time trends in mortality and disability-adjusted life years (DALY) rates over the past 30 years. The equation is formulated as follows: Y = *α* + *β*X + *ε*. In this linear regression model, X denotes the calendar year, Y represents the natural logarithm of the mortality and DALY rates, and ε signifies the error term. The EAPC is calculated as 100 × (exp(β) - 1). Additionally, we calculated the relevant 95% confidence interval (CI) using the aforementioned linear regression model. A 95% CI below 0 indicates a downward trend, while a 95% CI above 0 suggests an upward trend. A 95% CI that includes zero signifies a stable trend. We employed Pearson correlation coefficient analysis to examine the relationship between EAPC and the Socio-Demographic Index (SDI). Based on the quartiles of SDI values, countries and regions were categorized into low, low-middle, middle, high-middle, and high SDI groups, with values ranging from 0 to 1. We utilized the Bayesian Age-Period-Cohort (BAPC) model to predict disease burden trends over the next 20 years. The BAPC model is expressed as nij = log(λij) = *μ* + αi + βj + γk, where λij represents the number of cases, μ denotes the intercept, and αi, βj, and γk represent the effects of age, period, and cohort, respectively. All data analyses and visualizations presented in this article were conducted using the open-source software R (version 4.4.1) and JD_GBDR (V2.26, Jingding Medical Technology Co., LTD.).

## 3 Results

### 3.1 The global burden of tobacco-related Alzheimer’s disease over 55 years of age in 2021

The global burden of tobacco-related Alzheimer’s disease among individuals over 55 years of age in 2021 revealed an estimated 29,139 tobacco-related deaths and 839,802.32 disability-adjusted life years (DALY), with respective 95% uncertainty intervals (UI) of 3,308–59,819 and 94,015.59–1,635,935.91. The mortality and ASDRs were calculated at 1.96% (95% UI, 0.22–4.03) and 56.51% (95% UI, 6.33–110.09), respectively. In comparison, in 1990, the number of tobacco-related deaths and DALY for individuals over 55 years old were 31,688 (95% UI, 3,756–65,393) and 1,000,996.74 (95% UI, 116,170.11–1,915,328.53), both of which were higher than those recorded in 2021. This trend indicates a decrease in the number of deaths and DALY over the past 30 years (Table 1; Supplementary Table 1).

**Table 1:** The death cases and rates of tobacco-related AD among individuals over the age of 55 in 1990 and 2021 and its EAPC.

Location	1990		2021		1990–2021 EAPC (95% CI)
	Deaths Case (95% UI)	Rate	Deaths Case (95% UI)	Rate	
Global	31687.71 (3756.19,65393.12)	4.72 (0.56,9.74)	29139.13 (3307.56,59819.32)	1.96 (0.22,4.03)	−2.92 (−3.00,-2.85)
Southeast Asia	5010.72 (591.41,9507.69)	11.83 (1.40,22.45)	5543.81 (680.27,10563.14)	4.84 (0.59,9.22)	−3.08 (−3.32,-2.85)
Oceania	71.08 (7.10,148.38)	14.77 (1.48,30.84)	111.21 (10.46,236.19)	9.01 (0.85,19.14)	−1.81 (−1.93,-1.69)
East Asia	4403.11 (536.26,8719.60)	2.96 (0.36,5.85)	3101.74 (343.17,6030.46)	0.79 (0.09,1.54)	−4.36 (−4.55,-4.17)
Central Europe	773.37 (90.62,1481.13)	2.92 (0.34,5.58)	153.41 (17.38,312.74)	0.41 (0.05,0.84)	−6.98 (−7.49,-6.46)
Australasia	76.09 (8.53,157.85)	1.93 (0.22,4.01)	27.29 (2.88,64.37)	0.31 (0.03,0.73)	−6.09 (−7.03,-5.15)
Central Asia	315.26 (35.86,625.61)	3.94 (0.45,7.82)	206.16 (24.36,410.18)	1.42 (0.17,2.82)	−3.95 (−4.46,-3.44)
High-income Asia Pacific	1404.51 (159.27,2692.71)	4.02 (0.46,7.70)	272.87 (28.97,586.86)	0.39 (0.04,0.83)	−8.99 (−9.57,-8.40)
Eastern Europe	956.27 (124.12,1757.81)	1.96 (0.25,3.60)	100.08 (13.26,193.04)	0.16 (0.02,0.31)	−9.53 (−10.23,-8.83)
High-income North America	458.93 (51.64,953.07)	0.79 (0.09,1.65)	243.08 (28.09,547.54)	0.22 (0.02,0.49)	−4.94 (−5.39,-4.48)
Western Europe	1899.02 (214.73,3793.61)	1.96 (0.22,3.91)	389.96 (43.46,874.04)	0.26 (0.03,0.59)	−7.00 (−7.64,-6.36)
Southern Latin America	83.12 (9.62,173.27)	1.05 (0.12,2.19)	40.86 (4.85,88.81)	0.28 (0.03,0.60)	−4.69 (−5.07,-4.30)
Caribbean	82.28 (9.74,169.11)	1.91 (0.23,3.92)	82.78 (9.49,175.65)	0.89 (0.10,1.90)	−2.76 (−3.00,-2.53)
Andean Latin America	26.04 (2.81,57.68)	0.78 (0.08,1.72)	17.40 (1.84,43.88)	0.18 (0.02,0.44)	−4.70 (−4.91,-4.49)
Central Latin America	291.00 (32.19,576.42)	2.14 (0.24,4.25)	96.70 (11.26,209.86)	0.23 (0.03,0.49)	−7.77 (−8.05,-7.49)
Tropical Latin America	218.77 (26.53,432.49)	1.44 (0.18,2.86)	158.56 (16.32,355.12)	0.36 (0.04,0.80)	−5.26 (−5.62,-4.90)
North Africa and Middle East	1591.42 (184.89,3128.17)	5.63 (0.65,11.07)	1486.93 (165.41,3009.23)	1.95 (0.22,3.95)	−3.62 (−3.74,-3.49)
South Asia	12690.22 (1405.92,30519.52)	13.37 (1.48,32.15)	15735.44 (1448.32,36696.98)	6.34 (0.58,14.78)	−2.23 (−2.34,-2.13)
Central Sub-Saharan Africa	126.44 (12.91,273.59)	3.36 (0.34,7.28)	154.92 (15.64,329.52)	1.72 (0.17,3.65)	−2.20 (−2.29,-2.11)
Eastern Sub-Saharan Africa	494.78 (55.67,1025.94)	4.07 (0.46,8.43)	486.96 (54.03,1012.70)	1.80 (0.20,3.75)	−2.80 (−2.89,-2.72)
Western Sub-Saharan Africa	356.92 (36.98,789.08)	2.47 (0.26,5.47)	354.78 (31.94,766.63)	1.10 (0.10,2.39)	−2.49 (−2.56,-2.43)
Southern Sub-Saharan Africa	358.34 (39.90,708.17)	8.10 (0.90,16.00)	374.19 (41.23,753.98)	3.84 (0.42,7.74)	−2.70 (−3.19,-2.21)
High SDI	4302.46 (510.58,8406.31)	2.31 (0.27,4.51)	1159.63 (136.26,2483.78)	0.34 (0.04,0.72)	−7.08 (−7.59,-6.56)
High-middle SDI	3636.24 (437.93,6798.28)	2.11 (0.25,3.94)	1933.53 (214.04,3697.05)	0.56 (0.06,1.07)	−4.85 (−5.09,-4.62)
Low SDI	3021.80 (330.85,6772.47)	8.10 (0.89,18.15)	3354.97 (363.32,7652.80)	4.09 (0.44,9.33)	−2.18 (−2.33,-2.02)
Low-middle SDI	12373.97 (1462.95,28206.43)	12.28 (1.45,27.98)	14792.06 (1471.66,32883.16)	6.14 (0.61,13.64)	−2.08 (−2.17,-1.99)
Middle SDI	8323.49 (978.43,15988.36)	4.80 (0.56,9.21)	7879.02 (919.91,15284.68)	1.68 (0.20,3.25)	−3.60 (−3.79,-3.42)

**Figure 1:**
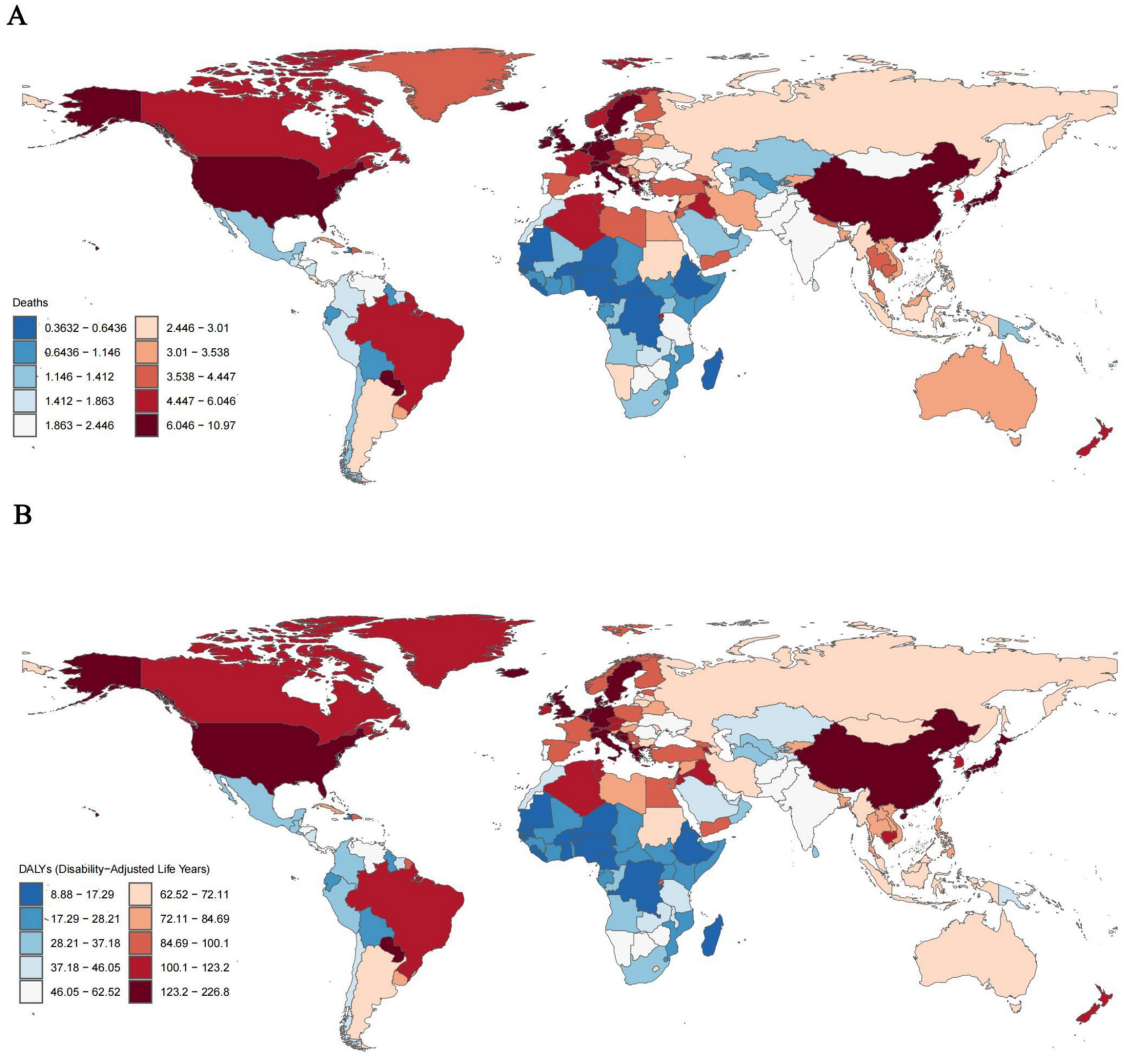
ASMRs **(A)** and ASDRs **(B)** of tobacco-related AD among individuals over the age of 55 globally, 2021.

Countries such as Lebanon, several European nations (including Greece, Denmark, Sweden, and the Netherlands), the United States, Japan, and China exhibited the highest rates of mortality and DALY. Specifically, Lebanon reported a tobacco-related AD mortality rate of 10.97% (95% UI, 2.73–30.24) and a DALY of 226.78% (95% UI, 96.60–505.54). Following Lebanon, China had a mortality rate of 6.49% (95% UI, 1.53–18.40) and a DALY of 152.63% (95% UI, 65.32–352.84) (Figures 1A,B).

The global mortality estimated annual percentage change (EAPC) for the tobacco-related AD burden among individuals over 55 years of age from 1990 to 2021 was −2.92 (95% UI, −3.00 to −2.85). By Global Burden of Disease (GBD) region, countries in West Asia (including Georgia, Lebanon, and Armenia) and Russia exhibited the highest mortality EAPC, with the corresponding age-standardized rates (ASR) also being the highest (Georgia EAPC: 2.81; Lebanon EAPC: 2.72; Armenia EAPC: 2.43; Russia EAPC: 1.64). Conversely, South Africa, South America, the United Arab Emirates, and other regions reported the lowest EAPC, which corresponded to the lowest ASR (South Africa EAPC: −3.67; Madagascar EAPC: −3.64; Grenada EAPC: −3.21; United Arab Emirates EAPC: −3.05) (Supplementary Figure 1).

Disaggregated by age, the global ASMR and ASDR related to tobacco and Alzheimer’s disease in individuals aged 55 years and older in 2021 were highest among those aged 95 years and older, accounting for 40% of the total (ASMR: 69.41, 95% UI, 17.00–182.41; ASDR: 694.90, 95% UI, 251.21–1621.53). This was followed by the age group of 90–94 years (ASMR: 50.24, 95% UI, 11.83–131.92; ASDR: 573.87, 95% UI, 221.65–1290.55). Overall, within the population over 55 years old, there was a general positive correlation between age and both mortality and DALY ratios (Supplementary Figures 2A,B).

When disaggregated by sex, the burden of tobacco-related Alzheimer’s disease in men over 55 years of age in 2021 was significantly higher than in women, with the ASMR for men approximately three times that of women (Male: 6.59, 95% UI, 1.56–18.65; Female: 2.58, 95% UI, 0.62–6.82). The ASDR for males was 151.65% (95% UI, 63.99–353.94), which was also about three times higher than that for females at 52.31% (95% UI, 22.68–117.06) (see Supplementary Figures 3A,B).

### 3.2 Regional burden of tobacco-related Alzheimer’s disease over 55 years of age in 2021

By region, data indicated that in most of the 21 regions globally, the ASMR and ASDR for tobacco-related Alzheimer’s disease among individuals aged 55 years and older have decreased since 1990. However, the high-income Pacific Rim, Eastern Europe, and Central Asia have experienced increases in these rates since 1990, with mortality rates rising by 1.1, 0.1, and 0.05%, respectively (Supplementary Figures 4A,B).

Disaggregated by sex, East Asian men exhibited the highest tobacco-related Alzheimer’s mortality rate of 10.49% (95% UI, 2.52–30.78) and a DALY rate of 254.34 (95% UI, 106.49–600.59) among individuals over 55 years of age. This was followed by high-income North America, while West sub-Saharan Africa recorded the lowest rates, with a mortality rate of 0.87% (95% UI, 0.20–2.53) and a ASDR of 22.01% (95% UI, 8.55–53.88). Among women over 55 years of age, high-income North America reported the highest tobacco-related Alzheimer’s mortality and ASDRs, with an ASMR of 7.29% (95% UI, 1.80–18.89) and an ASDR of 146.51% (95% UI, 65.31–325.63), followed by Western Europe, Australia, while South Africa and Asia reported the lowest rates. Notably, the prevalence of tobacco-related Alzheimer’s mortality and ASDRs in women over 55 years exceeded that of men in Australia and high-income North America, with Australia showing the most pronounced difference, where the mortality rate was 4.62% (95% UI, 1.13–12.38). This figure was significantly higher than the local male mortality rate of 2.86% (95% UI, 0.64–8.38) (Supplementary Figures 3A,B).

### 3.3 Temporal trends in tobacco-related Alzheimer’s disease in people over 55 years of age from 1990 to 2021

In terms of age stratification, from 1990 to 2021, the global mortality rate and Disability-Adjusted Life Years (DALY) across all age groups over 55 years exhibited a decreasing trend. Notably, there was a decline in the mortality rate for individuals aged 90 to 94 years (2021 ASMR: 50.24, 95% UI: 11.83–131.92; 1990 ASMR: 68.96, 95% UI: 15.94–186.64). The age group of those over 95 years showed the most significant decrease (2021 ASMR, 69.41, 95% UI: 17.00–182.41; 1990 ASMR: 107.03, 95% UI: 25.08–282.26) (Supplementary Figures 3C,D).

**Figure 2:**
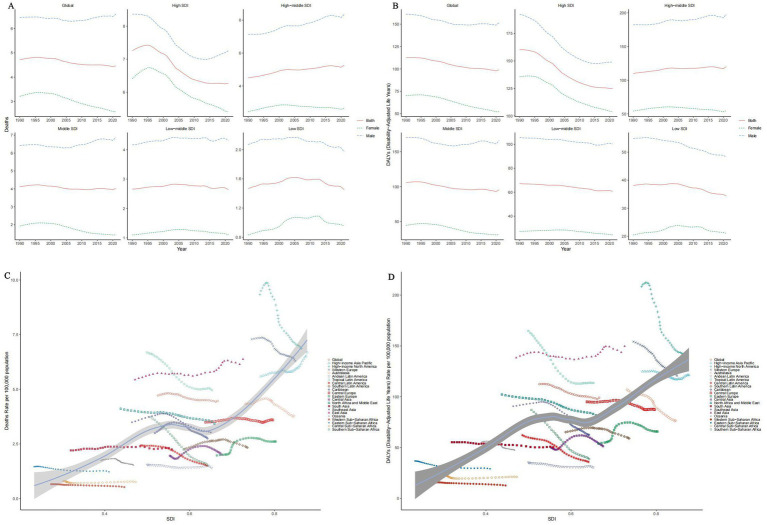
Temporal trends of ASMRs **(A)** and ASDRs **(B)** of tobacco-related AD among individuals over the age of 55 by SDI, 1990–2021. Temporal trends of ASMRs **(C)** and ASDRs **(D)** of tobacco-related AD among individuals over the age of 55 by SDI and region, 1990–2021.

By region, the global mortality rate and DALY demonstrated a slight overall decline from 1990 to 2021. Following a brief increase from 1990 to 1995 (1995 ASMR: 7.43, 95% UI: 1.75–20.92), countries with high Socio-Demographic Index (SDI) experienced a significant decline starting in 1996, although their rates remained above the global average (2021 ASMR: 6.27, 95% UI: 1.48–17.29). In contrast, male tobacco ASMRs in middle and low SDI regions have slightly increased, with 2021 ASMRs of 4.03 (95% UI: 0.95–11.35) for middle SDI and 2.64 (95% UI: 0.60–7.54) for low-middle SDI regions, respectively (Figures 2A,B).

When stratified by SDI, a positive correlation between SDI and both mortality and DALY was observed overall. Among the 21 Global Burden of Disease (GBD) regions, mortality decreased in most regions with increasing SDI; however, some regions, including the high-income Pacific Rim, East Asia, and Eastern Europe, exhibited either an increase or fluctuation in mortality rates (Figures 2C,D).

### 3.4 Future trends in the burden of tobacco-related Alzheimer’s disease in people over 55 years of age

**Figure 3:**
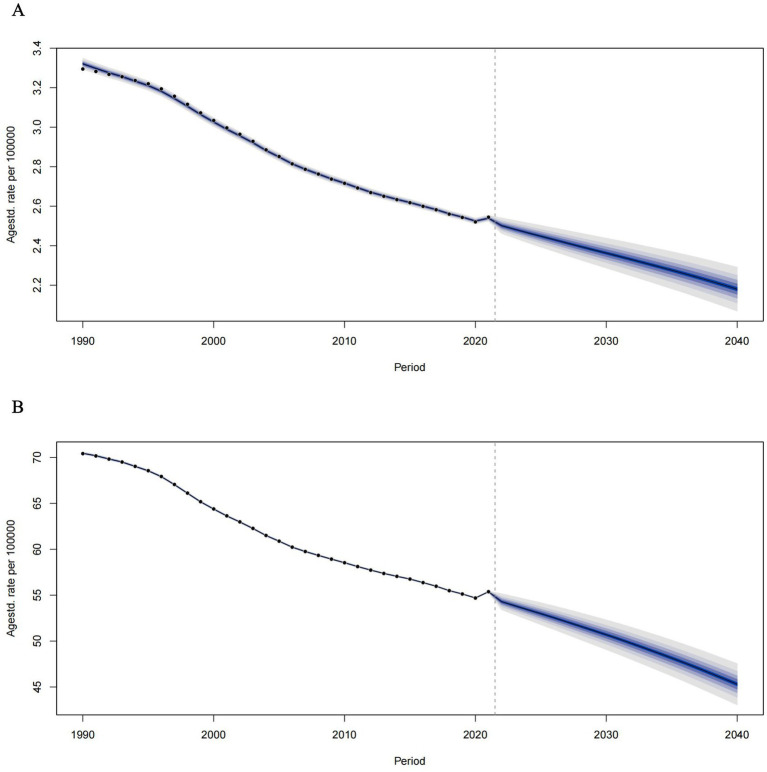
Temporal trends of ASMRs **(A)** and ASDRs **(B)** of tobacco-related AD among individuals over the age of 55 from 1990 to 2021 and prediction for future trends from 2021–2040.

According to estimates derived from the Bayesian Age-24 cohort (BAPC) model, the burden of tobacco-related Alzheimer’s disease among individuals over the age of 55 is anticipated to gradually decline. By 2040, the global age-standardized ASMR (ASMR) for Alzheimer’s disease in this demographic is projected to decrease from 2.18 per 100,000 in 2021 to 0.87 per 100,000 in 2040. Additionally, the age-standardized rate of Disability-Adjusted Life Years (DALYs) is expected to fall from 55.38 per 100,000 in 2021 to 19.58 per 100,000 in 2040 (see Figure 3).

## 4 Discussion

This article presents the latest study on the global burden of tobacco-related Alzheimer’s disease (AD) among individuals aged 55 and older, covering the period from 1990 to 2021. The study indicated a relative reduction in the burden of tobacco-related AD in this demographic in 2021, with estimated deaths and disability-adjusted life years (DALY) amounting to 29,139 (95% UI, 3,307–59,819) and 839,802.32 (95% UI, 94,015.59–1,635,935.91), respectively, compared to 1990. However, the burden of tobacco-related AD exhibits significant variation across regions, age groups, genders, and socio-demographic indices (SDI). These findings may assist governments in formulating relevant regional regulations.

Tobacco is a well-documented risk factor for numerous health issues, with extensive international research linking it to the onset of various diseases, including major non-communicable diseases such as chronic obstructive pulmonary disease (COPD), hypertension, and cancer (13–15). In 2008, the World Health Organization (WHO) addressed global smoking rates and their implications for public health, emphasizing the urgent need for tobacco control measures (4).

Although nicotine, the primary component of tobacco, acts as an agonist to up-regulate nicotinic acetylcholine receptors, thereby enhancing cognitive function and attention in the short term (16), long-term tobacco use can lead to accelerated brain atrophy, decreased perfusion, white matter lesions, and a reduction in neurofibrillary tangles (17). As an agonist of the nicotinic acetylcholine receptor (particularly the α4β2 and α7 subtypes), nicotine enhances cholinergic signaling in the short term. However, prolonged exposure leads to receptor desensitization (decreased function) and compensatory upregulation (increased receptor numbers), ultimately disrupting cholinergic homeostasis (18). Long-term nicotine exposure may exacerbate apoptosis in cholinergic neurons due to oxidative stress and mitochondrial dysfunction, particularly affecting the basal forebrain, which is the primary distribution area of cholinergic neurons in AD (19). Regarding the mechanisms by which nicotine may worsen AD, its activation of α7nAChR could potentially increase the toxicity of Aβ oligomers or interfere with the clearance of Aβ (20). Nicotine may activate kinases such as GSK-3β, promoting the hyperphosphorylation of Tau protein (21). Long-term exposure to nicotine can lead to dysfunction of nicotinic acetylcholine receptors, impairing long-term potentiation and adversely affecting learning and memory (22). Tobacco smoke contains a significant number of free radicals, including hydrogen peroxide, which can cause mitochondrial DNA damage and disrupt neuronal energy metabolism (23). Additionally, tobacco activates microglia, leading to the release of pro-inflammatory factors such as Interleukin-6 (IL-6) and Tumor Necrosis Factor-*α* (TNF-α), thereby exacerbating neuroinflammation (24). Smoking also induces cerebral vascular endothelial dysfunction, reduces cerebral blood flow, and exacerbates ischemic injury in AD (25). In conclusion, tobacco may synergistically promote the development of AD through multiple pathways, including cholinergic injury, Aβ/Tau pathology, oxidative stress, neuroinflammation, and vascular injury. While the global burden of tobacco-related AD is expected to decline over the next 20 years, it is suspected that this decline may slow. If the mechanism of cholinergic injury is further substantiated, this trend may rebound regionally in areas with poor implementation of tobacco control policies. However, more precise evidence is required, and further research is needed to explore the mechanisms of nicotine-induced cholinergic damage and its long-term effects on cognition.

The mental, caregiving, and economic burdens that each Alzheimer’s patient imposes on their family are substantial, while the overall economic impact on society is astronomical. Middle-aged and older adults individuals, who could otherwise continue contributing to society, often find themselves unable to care for themselves after developing this disease, thus becoming a burden to their relatives. They may experience disorientation when out in public, necessitating the use of financial and social resources for assistance. As many countries face aging populations, the prevalence of Alzheimer’s disease is expected to rise. Our study indicated that the contribution of tobacco to the burden of Alzheimer’s disease is likely to increase with higher Social Development Index (SDI) levels as local economies develop. The combination of these two factors suggests that the societal pressure of Alzheimer’s disease will intensify. This underscores the importance of government efforts to reduce smoking rates.

Although some governments worldwide have implemented policies to restrict smoking—such as increasing tobacco taxes and banning tobacco advertising (26)—the actual impact on the disease burden of Alzheimer’s disease (AD) remained unsatisfactory. For instance, in 2021, the United States and China reported the highest tobacco-related AD mortality rates among individuals over the age of 55, whereas both of the countries had certain limitations on cigarettes. This suggests that additional effective tobacco control measures are necessary to improve the current situation. Such measures could include enhancing public education about the health hazards of tobacco, enforcing strict smoking bans in public spaces, encouraging the use of e-cigarettes to substitute traditional cigarettes, and organizing community-based smoking cessation initiatives aimed at social welfare to better reduce smoking rates.

Regarding gender differences, the global tobacco-related AD ASMR among men over 55 years of age in 2021 was three times higher than that of women, indicating a greater disease burden for men. An analysis of the Socio-Demographic Index (SDI) from 1990 to 2020 reveals a continuous rise in the tobacco-related AD mortality rate among men over 55 in middle and high SDI regions, with rates in East Asia and the Pacific Rim significantly exceeding the global average. This trend may be attributed to the social division of labor, where men often bear the primary burden of economic development. To alleviate work-related stress, smoking has become a prevalent coping mechanism, leading to long-term neurological damage (27). In these regions, promoting healthier stress-relief alternatives—such as outdoor sports, social interactions, and engaging in leisure activities— and smoke-free workplace program may potentially mitigate the disease burden associated with tobacco use.

Our study revealed that in Australia and North America, tobacco-related Alzheimer’s disease (AD) ASMRs and disability-adjusted life years (DALY) rates for women over 55 years of age surpassed those for men. This trend may be attributed to the influence of the social and cultural climate since the 21st century, where women in both regions were more likely to encounter tobacco exposure in early adulthood, leading to a higher propensity for developing long-term smoking habits, a phenomenon that was less prevalent in the late 20th century (26). The detrimental effects of tobacco on human health can be enduring as recent research indicated that the impact of smoking on the immune system may persist for over a decade (28). In contrast, South Africa, Central Asia, and South Asia exhibited lower mortality rates, which may be attributed to relatively underdeveloped economic conditions in the former, and the promotion of smokeless tobacco and other policies in the latter, resulting in a social atmosphere where women were less exposed to tobacco (29). This highlighted the concerning trend of a ‘tobacco culture’ that has been increasingly compromising women’s health.

In summary, our study demonstrated that the tobacco-related burden of Alzheimer’s disease in individuals over 55 years of age varied significantly by region, country, age, sex, and socio-demographic index (SDI). For regions and countries with a heightened burden of tobacco-related AD among those over 55, there was an urgent need for governments to consider region-specific interventions, such as stricter tobacco control in high-SDI countries and gender-targeted cessation programs in regions like Australia where female AD burden exceeds males, thereby alleviating the health impacts of tobacco in later life.

This study has several shortcomings. First, the extent of group tobacco exposure, including both the frequency and quantity of smoking as well as second-hand smoke exposure among the non-smoking population, remains unknown. This limitation may result in an analysis that is not sufficiently comprehensive and introduces certain biases. Second, since the data is sourced from the Global Burden of Disease (GBD) database, variations in methodological standards for collecting tobacco medical history across different regions may exist. Factors such as population selection, sample size, and the design of the questionnaires may contribute to data discrepancies. Third, while the GBD 2019 study offers valuable and reliable estimates of disease burden, it lacks data on the prevalence and incidence of tobacco-related Alzheimer’s disease, preventing a more in-depth analysis.

## 5 Conclusion

Despite the fact that the tobacco-related burden of Alzheimer’s disease in middle-aged and older adults declined globally from 1990 to 2021 and is expected to continue to decline over the next 20 years, significant disparities existed across regions, age groups, genders, and Socio-Demographic Index (SDI). There was a surging tendency of tobacco-related AD burden with aging. The study illustrated a significantly higher burden in men than women, especially East Asian men. The burden was particularly evident in certain European and Western Asian countries, Australian women, and countries with High SDI. These data stressed the role of tobacco in AD patients and may assist governments in formulating relevant policies.

## Data Availability

The original contributions presented in the study are included in the article/Supplementary material, further inquiries can be directed to the corresponding author.

## Footnotes

^1^ http://ghdx.healthdata.org/gbd-results-tool
