# Supplementary material for: Global, regional, and national disease burden of tobacco-related Alzheimer’s disease among individuals over the age of 55: a global burden of disease study
Source: Front Public Health. 2025 May 14;13:1581871. doi: 10.3389/fpubh.2025.1581871 (PMC12116306; doi:10.3389/fpubh.2025.1581871)
Supplement: Supplementary file 1 [file Data_Sheet_1.docx]

**Supplementary Tables/figures**

Supplementary Table 1. The DALY number and rates of tobacco-related AD among individuals over the age of 55 in 1990 and 2021 and its EAPC

Supplementary Figure 1. The global mortality estimated annual percentage change (EAPC) for the tobacco-related AD burden among individuals over 55 years of age globally from 1990 to 2021

Supplementary Figure 2. ASMR(A) and ASDR(B) of tobacco-related AD among individuals over the age of 55 by region and age, 2021. Temporal trends of ASMR(C) and ASDR(D) of tobacco-related AD among individuals over the age of 55 by age, 1990-2021

Supplementary Figure 3. ASMRs(A) and ASDRs(B) of tobacco-related AD among individuals over the age of 55 by region and sex, 2021

Supplementary Figure 4. Temporal trends of ASMR(A) and ASDR(B) of tobacco-related AD among individuals over the age of 55 by region, 1990-2021

Supplementary Table 1.The DALY number and rates of tobacco-related AD among individuals over the age of 55 in 1990 and 2021 and its EAPC


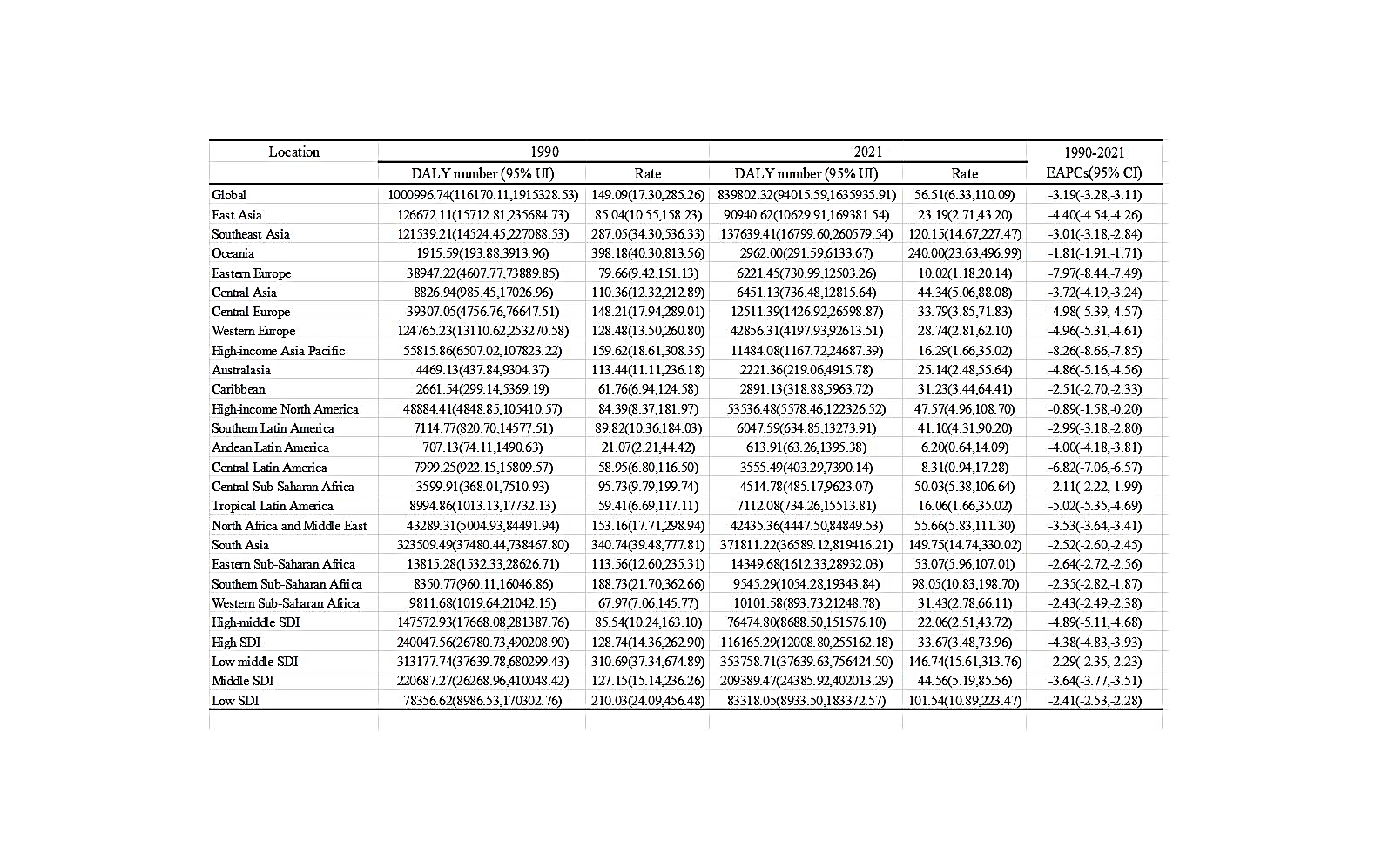


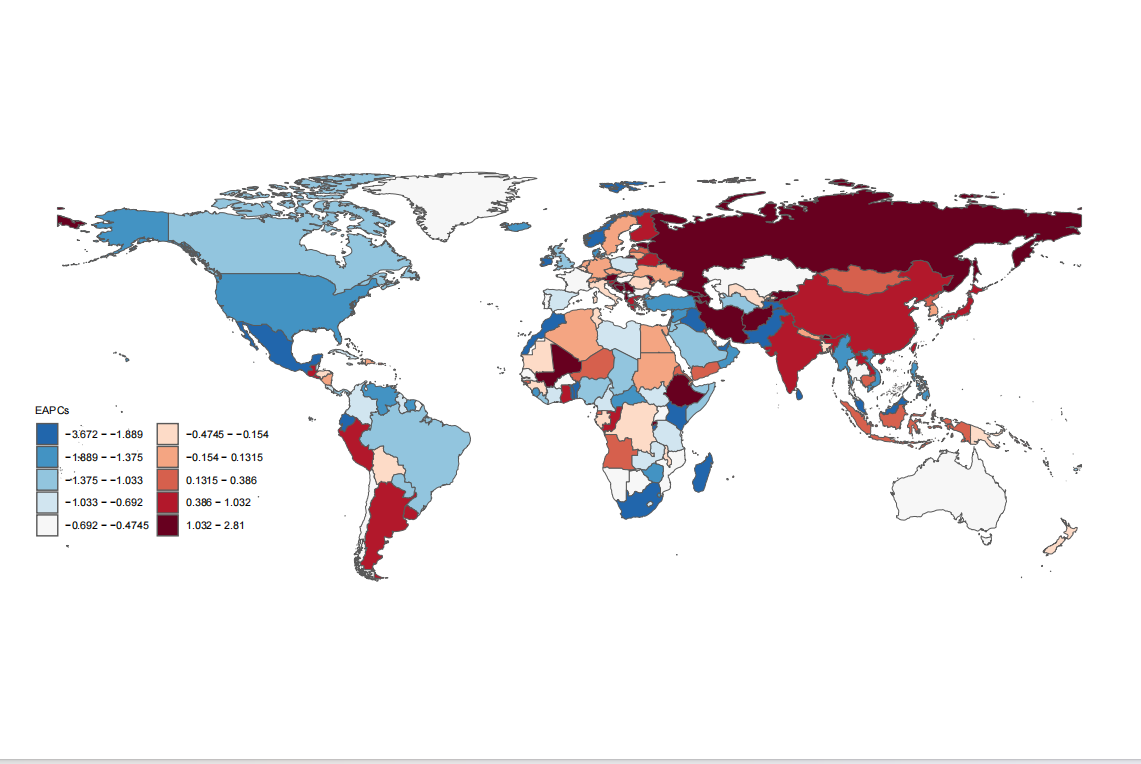


Supplementary Figure 1. The global mortality estimated annual percentage change (EAPC) for the tobacco-related AD burden among individuals over 55 years of age globally from 1990 to 2021

Alt text: A map demonstrated the global mortality estimated annual percentage change (EAPC) for the tobacco-related AD burden among individuals over 55 years of age globally from 1990 to 2021


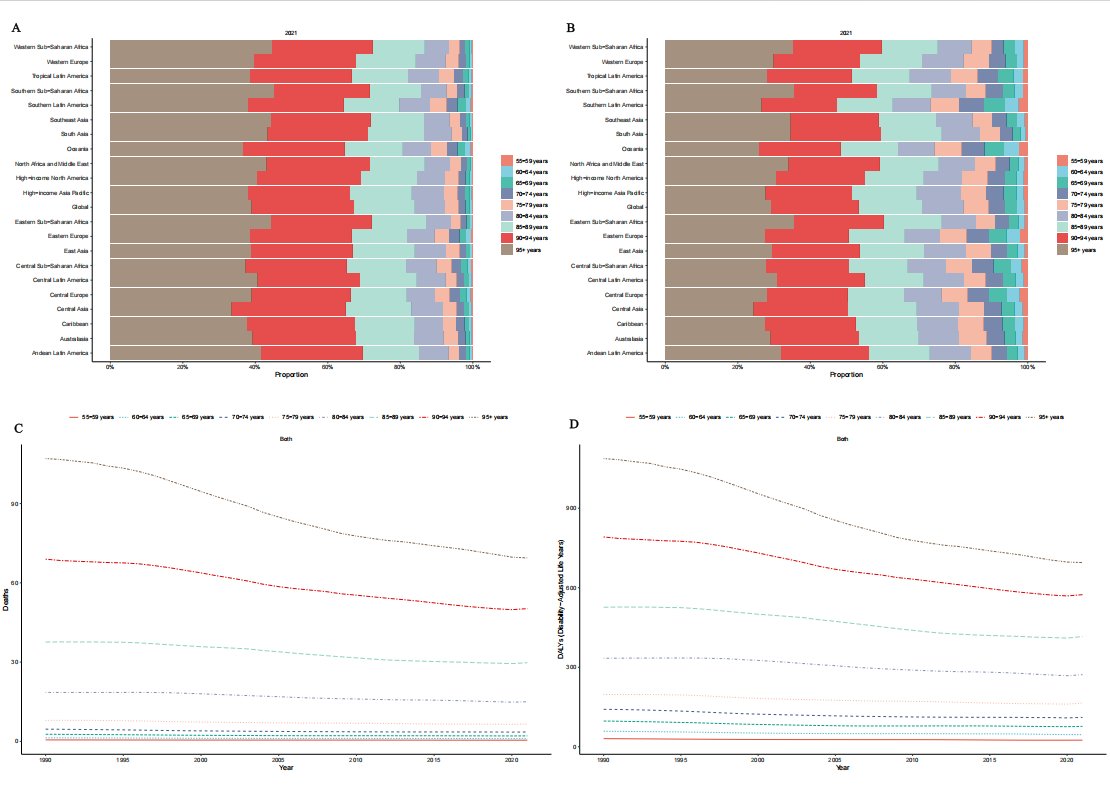


Supplementary Figure 2. ASMR(A) and ASDR(B) of tobacco-related AD among individuals over the age of 55 by region and age, 2021. Temporal trends of ASMR(C) and ASDR(D) of tobacco-related AD among individuals over the age of 55 by age, 1990-2021

Alt text: Two bar graphs showed ASMR(graph A) and ASDR(graph B) of tobacco-related AD among individuals over the age of 55 by region and age, 2021. Two line charts illustrated temporal trends of ASMR(graph C) and ASDR(graph D) of tobacco-related AD among individuals over the age of 55 by age, 1990-2021


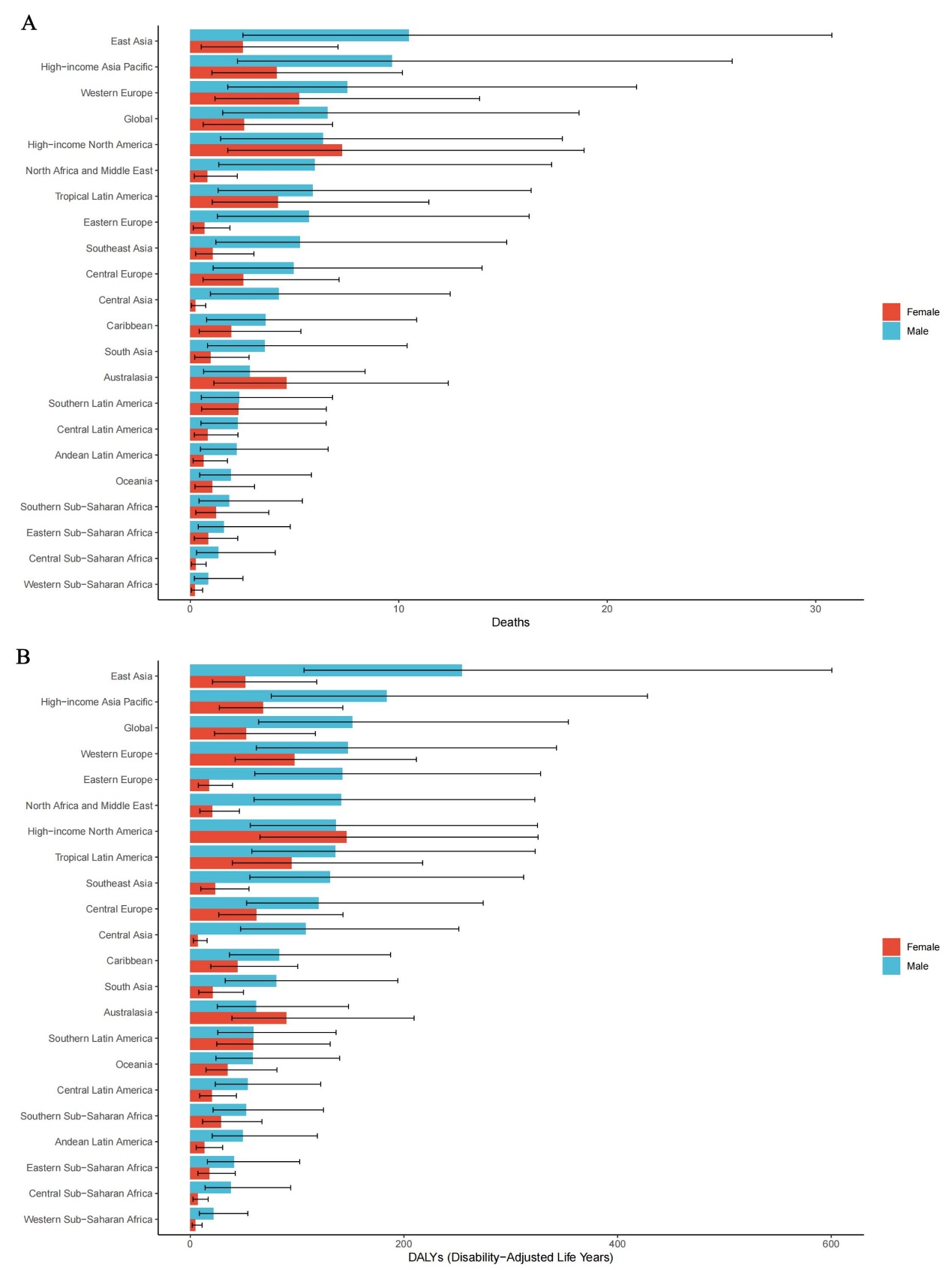


Supplementary Figure 3. ASMRs(A) and ASDRs(B) of tobacco-related AD among individuals over the age of 55 by region and sex, 2021

Alt text: Two bar charts demonstrated ASMR(Chart A) and ASDR(Chart B) of tobacco-related AD among individuals over the age of 55 by region and sex, 2021


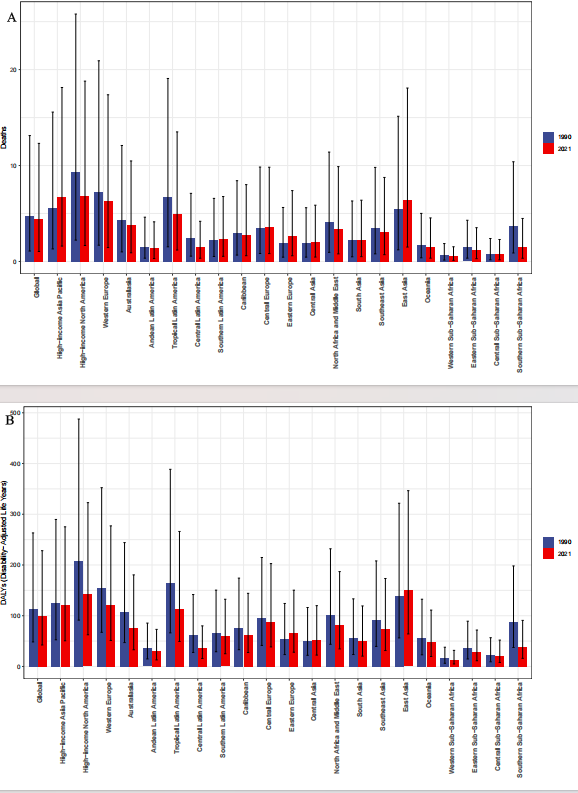


Supplementary Figure 4. Temporal trends of ASMR(A) and ASDR(B) of tobacco-related AD among individuals over the age of 55 by region, 1990-2021

Alt Text: Two bar graphs demonstrated temporal trends of ASMR(graph A) and ASDR(graph B) of tobacco-related AD among individuals over the age of 55 by region, 1990-2021

**Abbreviations:**

1. AD - Alzheimer's Disease
2. DALYs - Disability-Adjusted Life Years
3. EAPCs - Estimated Annual Percentage Changes
4. GBD - Global Burden of Disease
5. ASDRs - Age-Standardized Disability Rates
6. ASMR - Age-Standardized Mortality Rate
7. SDI - Socio-Demographic Index
8. ICD - International Classification of Diseases
9. CRA - Comparative Risk Assessment
10. CI - Confidence Interval
11. BAPC - Bayesian Age-Period-Cohort
12. COPD - Chronic Obstructive Pulmonary Disease

**Socio-demographic index (SDI)**

SDI was originally constructed for GBD 2015 by using the Human Development Index (HDI) methodology, wherein a 0 to 1 index value was determined for each of the original three covariate inputs (TFR in ages 15 to 49 years, EDU15+, and LDI per capita) by using the observed minima and maxima over the estimation period to set the scales. In response to feedback from collaborators and the evolution of the GBD, we have refined the indicator with each GBD cycle. Beginning in GBD 2017, along with our expanded estimation of age‐specific fertility, we replaced TFR with TFU25 as one of the three component indices. The TFU25 provides a better measure of women’s status in society because it focuses on ages at which childbearing disrupts the pursuit of education and entrance into the workforce. In addition, we observed that in highly developed countries, the TFU25 has tended to decline consistently over time despite rebounds in TFR driven by increasing fertility at older ages. Thus, for each covariate input, an index score of 0 represents the minimum level of each covariate input past which selected health outcomes can get no worse, and an indexscore of 1 represents the maximum level of each covariate input past which selected health outcomes cease to improve. As a composite, a ocation with an SDI of 0 would have a theoretical minimum level of sociodemographic development relevant to these health outcomes, and a location with an SDI of 1 (before multiplying by 100 for reporting) would have a theoretical maximum level of sociodemographic development relevant to these health outcomes.

We computed the index scores underlying SDI as follows:


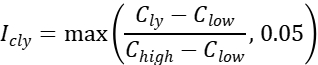


Where:
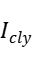
 is the index for covariate C, location l and year y and is equal to the difference between the value of this covariate in this location year and the lower limit of the covariate divided by the difference between the upper and lower limits for this covariate. For access to SDI information, please visit the public webpage at http://ghdx.healthdata.org/record/ihme-data/gbd-2019-socio-demographic-index-sdi-1950-2019

**Assessment of** **tobacco exposure**

Tobacco exposure is categorized as a subcategory of behavioral risk, defined as the current or previous active use of any tobacco product, including chewing tobacco and passive smoking. Exposure was measured by the number of cigarettes smoked per day and the cumulative years of smoking. In the social and human sciences, smoking exposure was assessed using self-reported data from major representative survey series that included relevant modules on household composition. These surveys comprise Demographic Health Surveys (DHS), Multiple Indicator Cluster Surveys (MICS), and Living Standards Measurement Surveys (LSMS), in addition to national and local censuses. Data sources also encompass the IPUMS project censuses, which are identified through the Global Health Data Exchange Directory (GHDx).

The Global Burden of Disease (GBD) study employed a conceptual framework of comparative risk assessment to quantify the burden of Alzheimer's disease attributable to smoking. Comparative risk assessment tools are valuable as they integrate evidence from multiple sources, focus on a specific health impact factor, assess the relationship between that factor and expected health outcomes, and utilize attribution strategies to determine the extent to which a single cause contributes to an outcome influenced by multiple causes. To calculate the burden of Alzheimer's disease due to smoking, the GBD study compared this risk distribution with the expected risk of the theoretical minimum risk exposure level, based on a continuous risk-outcome curve derived from nonparametric Bayesian splines. This approach combined the provided smoking exposure values to estimate the population attribution fraction (PAF) for these outcomes. The number of cause-specific deaths from each risk factor was calculated by multiplying the estimated PAF by the total number of deaths from the disease at each level.

When forecasting the prevalence of different smoking groups, it is important to consider that current smokers have a higher relative risk of mortality than former smokers, who in turn have a higher risk than never smokers. While the reference scenario incorporates these differing mortality rates across smoking statuses by forecasting smoking prevalence in period space, differential mortality must be explicitly added to the alternative scenario prevalence forecasts. To account for the effects of different mortality rates between current, former, and never smokers, we estimated the Alzheimer’s disease relative risks of mortality by smoking status. We computed exposure-weighted relative risks by location, age, sex, and cause in 2022 and aggregated these cause specific relative risks across all causes to generate an Alzheimer’s disease relative risk of mortality. Finally, we computed the mortality rate among never smokers and used each of the mortality rates to adjust our prevalence estimates in every future year.

Using these inputs, we calculated smoking PAFs for every location, sex, 5-year age group, and cause at 5-year intervals between 2022 and 2040, using the formula below, adapted from the GBD study

PAF=
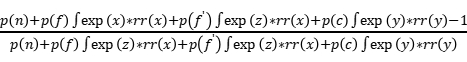


Where
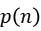
 is the prevalence of never smokers,
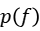
 is the prevalence of former smokers who quit in 2022 or earlier,
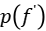
 is the prevalence of former smokers who quit in 2023 or later,
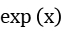
is a distribution of years since quitting among former smokers who quit in 2022 or earlier,
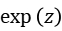
 is a uniform distribution of years since quitting among former smokers who quit in 2023 or later,
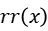
 is the relative risk for years since quitting,
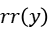
 is the prevalence of current smokers,
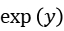
is a distribution of cigarettes per smoker per day or pack-years, and
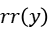
 is the relative risk for cigarettes per smoker per day or pack-years.

The Cause-of-death Integrated Model (CODEm) is an analytical tool developed from GBD data to estimate mortality and DALY for Alzheimer’s disease. This analysis focused on data collected in 1990 and 2021 to investigate trends in Alzheimer’s disease burden over this period. The selection of these years allows comprehensive comparisons to highlight changes in mortality and the overall impact of Alzheimer’s disease on global health over a nearly three-decade interval.

The fixed effects allow us to capture broad trends in both age patterns and the impacts of key biological and environmental covariates. The random effects allow for improved estimation by adding intercept shifts by GBD super-region, region, and country (the 187 countries are grouped into 21 regions based on both geographical proximity and epidemiologic similarity; the 21 regions are further grouped into seven more general super-regions), and changes in age patterns across regions and countries. The models of this family follow this form:


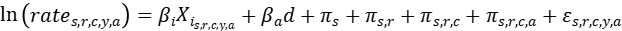


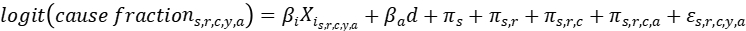


s = super-region index; r = region index; c = country index; y = year index; a = age index

[countries are nested within regions, which are nested within super-regions]


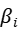
 = coefficient on covariate i


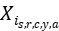
 = covariate i for observation s, r, c, y, a


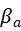
 = coefficient on age offsets


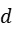
 = age dummy variables

π_s_ = random intercept on super-region

π_s, r_ = random intercept on region (nested within super-region)

π_s, r, a_ = random intercept on age (nested within region)

π_s, r, a, c_ = random intercept on country (nested within region-age)

Because of the small numbers that are often encountered for certain age groups, countries, or causes of death, covariate models may occasionally predict numbers that are negative in natural log or logit space. To avoid creating very large residuals that can negatively affect subsequent prediction steps, we have introduced a floor such that the predictions never go below a rate of .01 deaths per 100,000 people. In addition, log rate models, unlike logit cause-fraction models, are not constrained from predicting more deaths than the all-cause mortality rate. We have greater confidence in all-cause mortality predictions, because there are typically more data available for predicting all-cause mortality rates in the form of censuses, demographic and health surveys, vital registration systems that do not capture cause of death, etc. We have therefore placed a ceiling on log rate models such that they can never exceed the log all-cause mortality rate.

**YLLs and DALYs estimation process**

In GBD 2021, YLLs were calculated by multiplying the estimated number of deaths by the standard life expectancy at the age of death, stratified by age, sex, region, and year. To ensure accurate attribution of causes of death, GBD 2021 employed the principles of the 11th edition of the International Classification of Diseases (ICD-11), assigning each death to the underlying cause that initiated the chain of events leading to death. For deaths recorded with non-specific, unreliable, or intermediate cause codes, reallocation algorithms were applied to reassign these "garbage codes" to the most probable causes of death. These algorithms were derived from published studies, expert consultations, or regression-based adjustments using data from sources reporting multiple causes of death. The cause of death for most diseases and injuries is estimated using the Cause of Death Ensemble model (CODEm). CODEm employs a set of statistical models, systematically testing the predictive validity of different covariate combinations, then combining the results to estimate the number of deaths for specific causes by location, age, sex, and year. For a small number of causes with sparse data or significant changes in reporting practices, GBD 2021 adopted customized modeling strategies, including the use of prevalence, incidence, case fatality data, or data related to sub-causes to infer causes of death. Through this process, GBD 2021 achieved progress in controlling data heterogeneity and reducing uncertainty. To estimate DALYs in GBD 2021, specific cause mortality rates and YLDs were first estimated. DALYs for each year were then calculated by adding YLLs to YLDs. The uncertainty of YLLs was assumed to be independent of the uncertainty of YLDs. By summing the first set of YLLs and YLDs across 500 simulations, and repeating the process for subsequent simulations, the 95% uncertainty interval for DALYs was ultimately calculated. The estimation of DALYs covered every cause, location, age group, sex, and year, providing a comprehensive assessment of the global health burden.

**Data processing and heterogeneity control**

In GBD 2021, adjustments were made to epidemiological data known to have biases, such as those using alternative case definitions or measurement methods. These adjustments were made using correction factors estimated by MR-BRT (Meta-regression—Bayesian, regularized, trimmed), a collection of statistical models including linear and nonlinear mixed-effects models. The input data included paired estimates of two case definitions or measurement methods for the same age, sex, region, and year. MR-BRT also controlled for heterogeneity through network meta-regression and performed sex-splitting for inputs not reported by sex, and age-sex splitting for data not reported by either. These processes ensured data standardization and consistency, reducing heterogeneity issues caused by varying data sources and definitions. Additionally, input data spanning more than 25 years were disaggregated into finer age-specific estimates using alternative age patterns estimated from other available data sources.

**Method for data visualization**

| Name | Cite |
| --- | --- |
| bamp | Schmid V, Geressen F, Held L, Rainer E (2022). _ bamp: Bayesian Age-Period-Cohort Modeling and Prediction_. R package version 2.1.3, https://cran.r-project.org/web/packages/bamp/index.html. |
| data.table | Dowle M, Srinivasan A (2023). _data.table: Extension of `data.frame`_. R package version 1.14.8, <https://CRAN.R-project.org/package=data.table>. |
| digest | Lucas DEwcbA, Tuszynski J, Bengtsson H, Urbanek S, Frasca M, Lewis B, Stokely M, Muehleisen H, Murdoch D, Hester J, Wu W, Kou Q, Onkelinx T, Lang M, Simko V, Hornik K, Neal R, Bell K, de Queljoe M, Suruceanu I, Denney B, Schumacher D, Chang. aW (2022). _digest: Create Compact Hash Digests of R Objects_. R package version 0.6.31, <https://CRAN.R-project.org/package=digest>. |
| gglot2 | H. Wickham. ggplot2: Elegant Graphics for Data Analysis. Springer-Verlag New York, 2016. |
| dplyr | Wickham H, François R, Henry L, Müller K, Vaughan D (2023). _dplyr: A Grammar of Data Manipulation_. R package version 1.1.0, <https://CRAN.R-project.org/package=dplyr>. |
| forcats | Wickham H (2023). _forcats: Tools for Working with Categorical Variables (Factors)_. R package version 1.0.0, <https://CRAN.R-project.org/package=forcats>. |
| ggnewscale | Campitelli E (2022). _ggnewscale: Multiple Fill and Colour Scales in 'ggplot2'_. R package version 0.4.8, <https://CRAN.R-project.org/package=ggnewscale>. |
| ggrepel | Slowikowski K (2023). _ggrepel: Automatically Position Non-Overlapping Text Labels with 'ggplot2'_. R package version 0.9.3, <https://CRAN.R-project.org/package=ggrepel>. |
| ggsci | Xiao N (2023). _ggsci: Scientific Journal and Sci-Fi Themed Color Palettes for 'ggplot2'_. R package version 3.0.0, <https://CRAN.R-project.org/package=ggsci>. |
| inla | Krainski E T, Lindgren F, Rue H (2024). _INLAspacetime: Spatial and Spatio-Temporal Models using 'INLA'_. R package version 0.1.10, https://cran.r-project.org/web/packages/INLAspacetime/index.html |
| lubridate | Garrett Grolemund, Hadley Wickham (2011). _ Dates and Times Made Easy with lubridate. Journal of Statistical Software, 40(3), 1-25. URL <https://www.jstatsoft.org/v40/i03/>. |
| nordpred | Moller B, Weedon-Fekjaer H (2019) ._ nordpred: Fit power5 and poisson Age-Period-Cohort models to calculate prediction of cancer incidence and mortality_. R package version 1.1, URL http://www.kreftregisteret.no/software/nordpred/. |
| patchwork | Pedersen T (2022). _patchwork: The Composer of Plots_. R package version 1.1.2, <https://CRAN.R-project.org/package=patchwork>. |
| purrr | Wickham H, Henry L (2023). _purrr: Functional Programming Tools_. R package version 1.0.1, <https://CRAN.R-project.org/package=purrr>. |
| RColorBrewer | Neuwirth E (2022). _RColorBrewer: ColorBrewer Palettes_. R package version 1.1-3, <https://CRAN.R-project.org/package=RColorBrewer>. |
| readr | Wickham H, Hester J, Bryan J (2023). _readr: Read Rectangular Text Data_. R package version 2.1.4, <https://CRAN.R-project.org/package=readr>. |
| readxl | Wickham H, Bryan J (2023). _readxl: Read Excel Files_. R package version 1.4.2, <https://CRAN.R-project.org/package=readxl>. |
| sf | Pebesma, E., 2018. Simple Features for R: Standardized Support for Spatial Vector Data. The R Journal 10 (1), 439-446, <https://doi.org/10.32614/RJ-2018-009> Pebesma, E., & Bivand, R. (2023). Spatial Data Science: With Applications in R (1st ed.). Chapman and Hall/CRC. <https://doi.org/10.1201/9780429459016> |
| snow | Tierney L, Rossini AJ, Li N, Sevcikova H (2021). _snow: Simple Network of Workstations_. R package version 0.4-4, <https://CRAN.R-project.org/package=snow>. |
| snowfall | Knaus J (2022). _snowfall: Easier Cluster Computing (Based on 'snow')_. R package version 1.84-6.2, <https://CRAN.R-project.org/package=snowfall>. |
| stringr | Wickham H (2022). _stringr: Simple, Consistent Wrappers for Common String Operations_. R package version 1.5.0, <https://CRAN.R-project.org/package=stringr>. |
| tibble | Müller K, Wickham H (2022). _tibble: Simple Data Frames_. R package version 3.1.8, <https://CRAN.R-project.org/package=tibble>. |
| tidyr | Wickham H, Vaughan D, Girlich M (2023). _tidyr: Tidy Messy Data_. R package version 1.3.0, <https://CRAN.R-project.org/package=tidyr>. |
| tidyverse | Wickham H, Averick M, Bryan J, Chang W, McGowan LD, François R, Grolemund G, Hayes A, Henry L, Hester J, Kuhn M, Pedersen TL, Miller E, Bache SM, Müller K, Ooms J, Robinson D, Seidel DP, Spinu V, Takahashi K, Vaughan D, Wilke C, Woo K, Yutani H (2019). “Welcome to the tidyverse.” _Journal of Open Source Software_, *4*(43), 1686. doi:10.21105/joss.01686 <https://doi.org/10.21105/joss.01686>. |
| viridis | Simon Garnier, Noam Ross, Robert Rudis, Antônio P. Camargo, Marco Sciaini, and Cédric Scherer (2021). Rvision - Colorblind-Friendly Color Maps for R. R package version 0.6.2 |
| viridisLite | Simon Garnier, Noam Ross, Robert Rudis, Antônio P. Camargo, Marco Sciaini, and Cédric Scherer (2022). Rvision - Colorblind-Friendly Color Maps for R. R package version 0.4.1 |

**SDI quintiles for countries estimated in GBD 2021**

| SDI quintile | Locations included based on SDI values in 2021 from GBD 2021 results |
| --- | --- |
| Low SDI（0.00-0.47） | Afghanistan, Benin, Burkina Faso, Burundi, Central African Republic, Chad, Cote d'lvoire, Democratic Republic of the Congo, Eritrea, Ethiopia, Gambia, Guinea, Guinea-Bissau, Haiti, Liberia, Madagascar, Malawi, Mali, Mozambique, Nepal, Niger, Pakistan, Papua New Guinea, Rwanda, Senegal, Sierra Leone, Solomon Islands, Somalia, South Sudan, Togo, Uganda, United Republic of Tanzania, Yemen |
| Low-middle SDI（0.47-0.62） | Angola, Bangladesh, Belize, Bhutan, Bolivia (Plurinational State of), Cabo Verde, Cambodia, Cameroon, Comoros, Congo, Democratic People's Republic of Korea, Djibouti, Dominican Republic, El Salvador, Eswatini, Ghana, Guatemala, Honduras, India, Kenya, Kiribati, Kyrgyzstan, Lao People's Democratic Republic, Lesotho, Maldives, Marshall Islands, Mauritania, Micronesia (Federated States of), Mongolia, Morocco, Myanmar, Nicaragua, Nigeria, Palestine, Sao Tome and Principe, Sudan, Tajikistan, Timor-Leste, Tuvalu, Vanuatu, Venezuela (Bolivarian Republic of), Zambia, Zimbabwe |
| Middle SDI（0.62-0.71） | Albania, Algeria, Armenia, Azerbaijan, Botswana, Brazil, China, Colombia, Costa Rica, Cuba, Ecuador, Egypt, Equatorial Guinea, Fiji, Gabon, Grenada, Guyana, Indonesia, Iran (Islamic Republic of), Iraq, Jamaica, Mexico, Namibia, Nauru, Panama, Paraguay, Peru, Philippines, Saint Lucia, Saint Vincent and the Grenadines, Samoa, South Africa, Suriname, Syrian Arab Republic, Thailand, Tokelau, Tonga, Tunisia, Turkmenistan, Uzbekistan, Viet Nam |
| High-middle SDI（0.71-0.81） | American Samoa, Antigua and Barbuda, Argentina, Bahamas, Bahrain, Barbados, Belarus, Bosnia and Herzegovina, Bulgaria, Chile, Cook Islands, Croatia, Dominica, Georgia, Greece, Greenland, Hungary, Israel, Italy, Jordan, Kazakhstan, Lebanon, Libya, Malaysia, Malta, Mauritius, Montenegro, Niue, North Macedonia, Northern Mariana Islands, Oman, Palau, Poland, Portugal, Republic of Moldova, Romania, Russian Federation, Saint Kitts and Nevis, Saudi Arabia, Serbia, Seychelles, Spain, Sri Lanka, Trinidad and Tobago, Turkey, Ukraine, United States Virgin Islands, Uruguay |
| High SDI（0.81-1.00） | Andorra, Australia, Austria, Belgium, Bermuda, Brunei Darussalam, Canada, Cyprus, Czechia, Denmark, Estonia, Finland, France, Germany, Guam, Iceland, Ireland, Japan, Kuwait, Latvia, Lithuania, uxembourg, Monaco, Netherlands, New Zealand, Norway, Puerto Rico, Qatar, Republic of Korea, San Marino, Singapore, Slovakia, Slovenia, Sweden, Switzerland, Taiwan (Province of China), United Arab Emirates, United Kingdom, United States of America |
